# Aging related decreases in NM myosin expression and contractility in a resistance vessel

**DOI:** 10.3389/fphys.2024.1411420

**Published:** 2024-05-14

**Authors:** Young Soo Han, Rishiraj Bandi, Matthew J. Fogarty, Gary C. Sieck, Frank V. Brozovich

**Affiliations:** Departments of Physiology and Biomedical Engineering and Cardiovascular Diseases, Mayo Clinic, Rochester, MN, United States

**Keywords:** vascular reactivity, MYPT1, NM myosin, NO, endothelial independent and dependent vasodilatation

## Abstract

**Introduction:** Vasodilatation in response to NO is a fundamental response of the vasculature, and during aging, the vasculature is characterized by an increase in stiffness and decrease in sensitivity to NO mediated vasodilatation. Vascular tone is regulated by the activation of smooth muscle and nonmuscle (NM) myosin, which are regulated by the activities of myosin light chain kinase (MLCK) and MLC phosphatase. MLC phosphatase is a trimeric enzyme with a catalytic subunit, myosin targeting subunit (MYPT1) and 20 kDa subunit of unknown function. Alternative mRNA splicing produces LZ+/LZ- MYPT1 isoforms and the relative expression of LZ+/LZ- MYPT1 determines the sensitivity to NO mediated vasodilatation. This study tested the hypothesis that aging is associated with changes in LZ+ MYPT1 and NM myosin expression, which alter vascular reactivity.

**Methods:** We determined MYPT1 and NM myosin expression, force and the sensitivity of both endothelial dependent and endothelial independent relaxation in tertiary mesenteric arteries of young (6mo) and elderly (24mo) Fischer344 rats.

**Results:** The data demonstrate that aging is associated with a decrease in both the expression of NM myosin and force, but LZ+ MYPT expression and the sensitivity to both endothelial dependent and independent vasodilatation did not change. Further, smooth muscle cell hypertrophy increases the thickness of the medial layer of smooth muscle with aging.

**Discussion:** The reduction of NM myosin may represent an aging associated compensatory mechanism to normalize the stiffness of resistance vessels in response to the increase in media thickness observed during aging.

## Introduction

Both cardiac and vascular dysfunction are associated with aging, and during aging, the vasculature is characterized by an increase in stiffness and decrease in sensitivity to NO mediated vasodilatation (Lakatta, 1990; 2003; Shinmura et al., 2011; North and Sinclair, 2012). Endothelial dysfunction is thought to produce the decrease in sensitivity to NO mediated vasodilatation (Vatner et al., 2021). However, data are contradictory with some studies reporting that aging is associated endothelial dysfunction (Barton et al., 1997; Lubomirov et al., 2021; Zhong et al., 2021) and other studies not finding aging-related endothelial dysfunction (Barton et al., 1997; Luttrell et al., 2020). During aging, some have suggested that there is endothelial dysfunction in larger conduit arteries, but not smaller conduit or resistance vessels (Vatner et al., 2021). However, investigators have demonstrated a decrease in sensitivity to ACh mediated vasodilatation in both mouse mesenteric (Zhong et al., 2021) and femoral arteries (Lubomirov et al., 2021). Further, the response of vascular smooth muscle to contractile agonists in aging is variable with decreased (Barton et al., 1997; Nicholson et al., 2022), increased (Barton et al., 1997; Lubomirov et al., 2016; 2021) and unchanged (Zhong et al., 2021) sensitivity reported. The disparity is both vessel dependent (Barton et al., 1997; Lubomirov et al., 2018; Luttrell et al., 2020; Valovič et al., 2023) and due to regional differences (distal vs. proximal) within the same vessel (Zhang et al., 2018).

The level of phosphorylation of the 20 kDa regulatory myosin light chain (MLC_20_) defines vascular tone (Barsotti et al., 1987), and MLC_20_ phosphorylation is regulated by the activities of myosin light chain kinase (MLCK) and MLC phosphatase (Hartshorne et al., 1998; Somlyo and Somlyo, 2003; Brozovich et al., 2016). MLCK is regulated by Ca^2+^-calmodulin (Ikebe and Hartshorne, 1985), but the majority of signaling pathway that regulate vascular tone either inhibit or activate MLC phosphatase (Trinkle-Mulcahy et al., 1995; Hartshorne et al., 1998; Somlyo and Somlyo, 2003; Brozovich et al., 2016). MLC phosphatase is a trimeric enzyme with a catalytic subunit, myosin targeting subunit (MYPT1) and 20 kDa subunit of unknown function (Hartshorne et al., 1998). Alternative mRNA splicing produces several MYPT1 isoforms (Dirksen et al., 2000; Payne et al., 2004; Zhang and Fisher, 2007; Shukla and Fisher, 2008) and exclusion/inclusion of exon24 has been demonstrated to produce LZ+/LZ- MYPT1 isoforms (Payne et al., 2004; Shukla and Fisher, 2008; Zheng et al., 2015; Reho et al., 2016).

NO mediated vasodilatation is a fundamental response of the vasculature (Furchgott, 1999), and protein kinase G (PKG) mediated activation of MLC phosphatase is dependent on the interaction of PKG with the LZ+ MYPT isoform (Surks et al., 1999; Surks and Mendelsohn, 2003; Given et al., 2007). The expression of a LZ+ MYPT1 isoform has been demonstrated to be necessary for NO/cGMP/PKG mediated activation of the MLC phosphatase (Huang et al., 2004; Yuen et al., 2014), and the sensitivity to NO mediated vasodilatation has been demonstrated to be defined by the relative expression of LZ+/LZ- MYPT1 (Khatri et al., 2001; Huang et al., 2004; Payne et al., 2006; Yuen et al., 2011; Reho et al., 2016). Further, in addition to smooth muscle (SM) myosin, nonmuscle (NM) myosin is expressed in smooth muscle and has been demonstrated to participate in smooth muscle contraction (Morano et al., 2000; Löfgren et al., 2003; Rhee et al., 2006; Yuen et al., 2009; Zhang and Gunst, 2017; Lubomirov et al., 2023); changes in NM myosin expression and NM myosin activation have been demonstrated to influence vascular tone (Morano et al., 2000; Löfgren et al., 2003; Rhee et al., 2006; Yuen et al., 2009; Lubomirov et al., 2023).

This study was designed to test the hypothesis that aging is associated with changes in LZ+ MYPT1 and NM myosin expression, which then alter vascular reactivity. MYPT1 and NM myosin expression was determined in both tertiary mesenteric arteries and the aorta of young (6mo) and elderly (24mo) Fischer344 rats. In addition, in tertiary mesenteric vessels, we assessed vascular reactivity by determining maximal force and the sensitivity of both endothelial dependent and endothelial independent relaxation.

## Methods

### Animals

The experimental protocol was approved by the Mayo Clinic Institutional Animal Care and Use Committee and conformed to the guidelines of the National Institutes of Health. Male Fischer344 rats were studied at 6mo and 24mo.

### Immunoblotting

As previously described (Given et al., 2007; Yuen et al., 2009; Konik et al., 2013; Degen et al., 2015), immunoblotting was used to define protein expression. Briefly, samples of aortic and tertiary mesenteric vessels were homogenized in SDS sample buffer and total protein extract was resolved by SDS-PAGE, with sample loading normalized to total protein calculated within each band calculated from the stain-free prescast gel (Biorad, Cat#64551870), as previously described (Schaible et al., 2016; Yap et al., 2020; Han et al., 2021). After SDS-PAGE, proteins were transferred onto an immunoblot membrane (Biorad Cat#1629177) and anti-MYPT1 (ab32519, Abcam), -LZ+ MYPT1 (Given et al., 2007), -smooth muscle myosin heavy chain (ab53219, Abcam) and -α smooth muscle actin (ab5694, Abcam) were used to visualize the proteins. The resulting immunoblots were scanned and analyzed using ImageLab software (Han et al., 2021), and protein expression was normalized for total protein (TP) as previously described (Schaible et al., 2016; Yap et al., 2020; Han et al., 2021).

### Two-dimensional PAGE

NM and SM myosin expression was determined using 2D SDS-PAGE as previously described (Yuen et al., 2009; Han and Brozovich, 2013; Konik et al., 2013). We have demonstrated that this technique resolves the nonphosphorylated and phosphorylated SM myosin light chain (SM LC) and NM myosin light chain (NM LC) as four distinct spots (Yuen et al., 2009; Han and Brozovich, 2013; Konik et al., 2013). Briefly, samples of aortic and tertiary mesenteric smooth muscle were manually homogenized in 2D gel extraction buffer (7M urea, 2M thiourea, 4% CHAPS, 1% 3–5.6 immobilized pH gradient (IPG) buffer and EDTA-free Protease Inhibitor and PhosStop Phosphatase Inhibitor (Roche, Indianapolis, Ind., USA). The homogenates were cleared of lipids and extraneous salts using the 2D gel clean up kit (GE Healthcare). The acidic halves of 13-cm IPG DryStrip gels (pH 3–5.6 NL) were rehydrated in the presence of suitable amounts of sample in rehydration buffer solution (7M urea, 2M thiourea, 2% CHAPS, 0.5% pH 3.5–5 IPG buffer, 0.002% bromophenol blue and 12 μM/ml Destreak Reagent) for at least 10 h in the ‘face-down’ mode on the Ettan IPG rehydration tray and then resolved by isoelectric focusing in the ‘face-up’ mode on an Ettan IPGphor III (GE Healthcare). Following isoelectric focusing, the gel strips were equilibrated in 6M urea, 50 mM Tris-HCl, pH 6.4, 30% glycerol, 2% (w/v) SDS and 0.002% bromophenol blue, first containing 130 mM DTT for 15 min and then containing 135 mM iodoacetamide for 15 min before undergoing sodium dodecyl sulfate polyacrylamide gel electrophoresis (SDS-PAGE) for protein separation by molecular weight using the Bis-Tris buffering system with 12% gels (29:1). Subsequently, resolved 2D SDS-PAGE gels were silver stained. Gels were scanned using a high-resolution digital scanner (EPSON Perfection V750 Pro), and the spots were quantified using ImageQuant TL software. The two spots closest to the anode (spots 1 & 2) represent the phosphorylated and nonphosphorylated NM LC and the two spots nearest the cathode (spots 3 & 4) represent the phosphorylated and nonphosphorylated SM LC. As previously described (Yuen et al., 2009), the expression of NM myosin is calculated as [(1 + 2)/(1 + 2+3 + 4)]x100%].

### Muscle mechanics in tertiary mesenteric arteries

Mechanical studies were conducted using protocols previously published protocols (Han and Brozovich, 2013). Briefly, for force recordings, isolated tertiary mesenteric preparations (100–200 μm in diameter; ∼2 mm in length) with an intact endothelium were mounted using wires (40 μm in diameter) on a DMT 4-channel myograph system (Mulvany and Halpern, 1977; 2018; Mulvany et al., 1982) and stretched to L_o_ (the length for maximal force) in the myograph chamber containing continuously oxygenated physiological saline solution (PSS in mM: 140 NaCl, 3.7 KCl, 2.5 CaCl_2_, 0.81 MgSO_4_, 1.19 KH_2_PO_4_, 0.03 EDTA, 5.5 Glucose, 25 HEPES, pH 7.4). Following stretching the preparation to L_o_, the vessels were allowed to equilibrate for 1 h, and then were stimulated to contract with 80 mM KCl depolarization (in mM: 64.5 NaCl, 80 KCl, 2.5 CaCl_2_, 0.81 MgSO_4_, 1.19 KH_2_PO_4_, 0.03 EDTA, 5.5 Glucose, 25 HEPES, pH 7.4). The initial response to KCl depolarization was maintained for 10–15 min, before the vessel was relaxed with PSS. Then, the vessels were depolarized with KCl and after the force reached a steady state, the dose-response relationship of force relaxation produced by acetylcholine (ACh; 10nM-10 μM) was determined. The preparation was transferred to PSS, and following another contraction with 80 mM KCl, the relaxation produced by the cell permeable cGMP analog, 8Br-cGMP (100 μM), was assessed. Mesenteric preparations isolated from the same animal were used for both mechanical and molecular studies.

### Quantification of vessel hypertrophy

The extent of vascular smooth muscle cell hypertrophy was determined from hematoxylin-eosin (HE) stained sections of tertiary mesenteric arteries as previously described (Lin et al., 2020). The vessels were embedded in paraffin and then thin sections (10 μm) were stained with H&E (Han et al., 2021). Photomicrographs were obtained, and for each slide, at least six fields per slide were analyzed. Quantification of the total smooth muscle cell number, the thickness of the media layer of smooth muscle cells as well as vessel diameter and cross-sectional area was performed using ImageJ analysis software (Version 1.49, NIH, Bethesda, MD).

### Statistical analysis

All data are presented as mean ± SEM (n = number of animals). In designing experiments, a power analysis was performed (power = 80%, α = 0.05) to determine the number of animals per group (6 animals per group). Differences between groups (6mo vs. 24mo) were compared using a two-way ANOVA, and if significant differences were found, a Student’s t-test was used *post hoc* to compare values with a *p* < 0.05 level significance.

## Results

**FIGURE 1:**
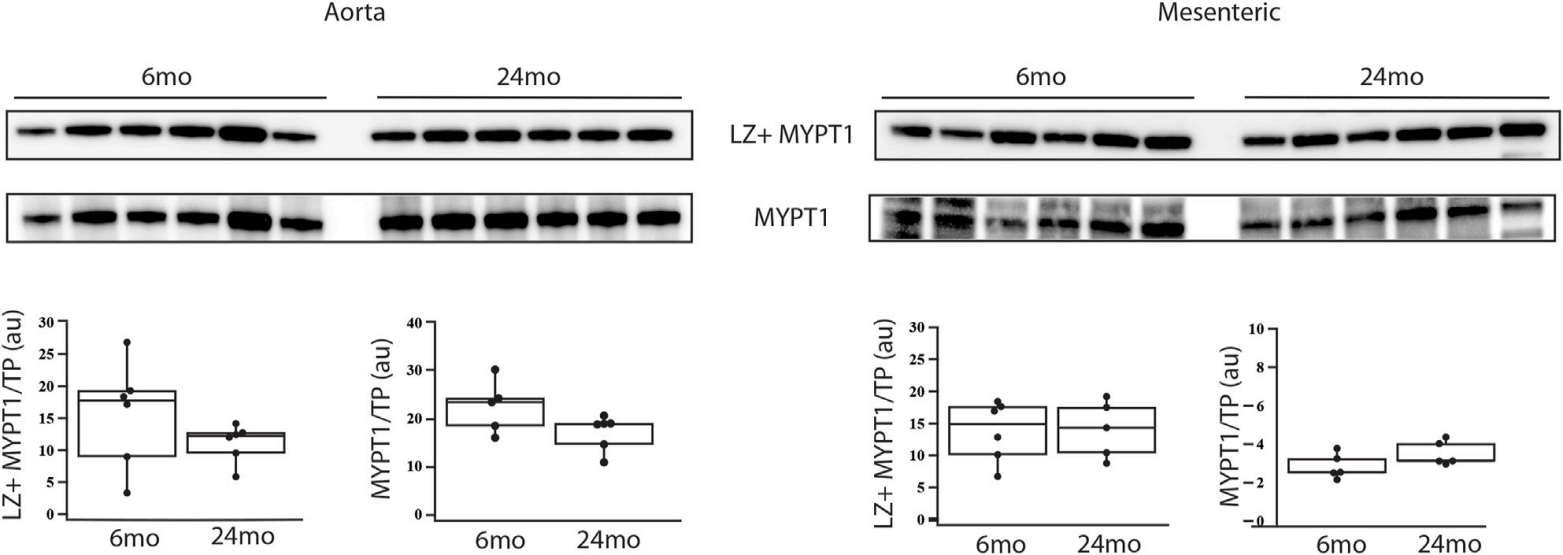
During aging, MYPT1 and LZ + MYPT1 expression does not change. Western blots of MYPT1 and LZ + MYPT1 expression in the aorta and tertiary mesenteric artery. MYPT1 and LZ + MYPT1 expression were normalized to total protein (TP) as previously described (Schaible et al., 2016; Yap et al., 2020; Han et al., 2021). Box blots summarize the data; neither MYPT1/TP nor LZ + MYPT1/TP expression is altered during aging, *p* > 0.05.

The expression of MYPT1 and LZ+ MYPT1 did not change with aging (6mo (n = 6) vs. 24mo (n = 6)). in either the aorta (Figure 1) or tertiary mesenteric arteries (Figure 1). In the aorta, MYPT1 expression normalized to total protein (MYPT1/TP) was 22.6 ± 2.2au vs. 18.5 ± 0.9au (*p* > 0.05) and LZ+ MYPT1/TP expression was 15.7 ± 3.4au vs. 11.1 ± 1.2au (*p* > 0.05). In the tertiary mesentery arteries, MYPT1/TP expression was 2.8 ± 0.3au vs. 3.5 ± 0.3au (*p* > 0.05), while MYPT1 LZ+/TP was 13.8 ± 1.9au vs. 14.1 ± 5.0au (*p* > 0.05).

**FIGURE 2:**
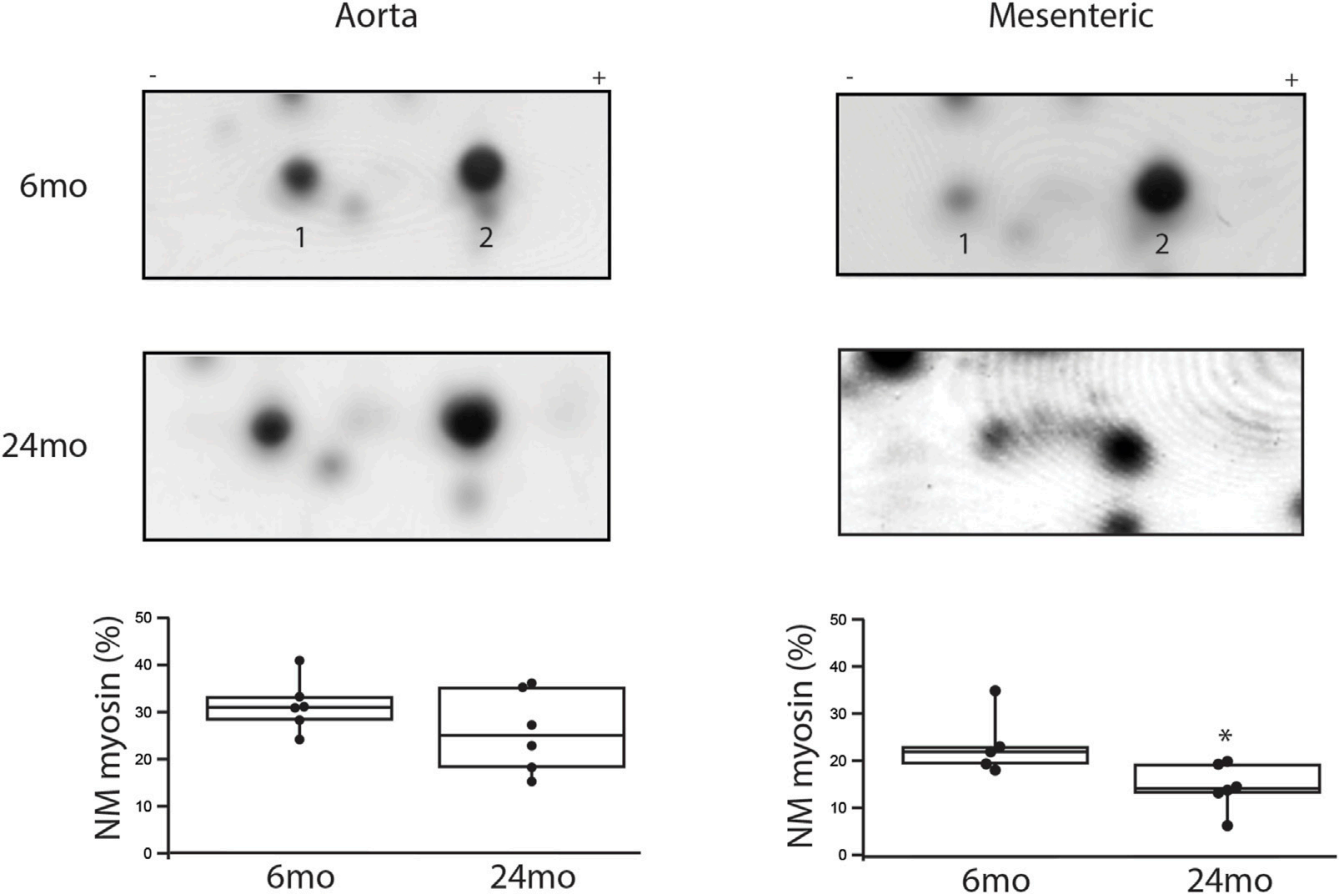
During aging, NM myosin expression decreases in a resistance, but not a conduit vessel. Two-dimensional SDS-PAGE was used to separate the NM myosin light chain (1) and SM light chain (2). The expression of NM myosin as a percentage of total myosin is the density of (1/(1 + 2))x100% (Yuen et al., 2009). Box plots summarize NM myosin expression; during aging, NM myosin does not change in the aorta (31% ± 2% vs. 26% ± 4%, *p* > 0.05), but declines significantly in the tertiary mesenteric artery (22% ± 3% vs. 12% ± 2%, *p* = 0.025); *, *p* < 0.05.

The expression of NM and SM myosin was assessed using 2-dimensional PAGE (Yuen et al., 2009; Han and Brozovich, 2013; Konik et al., 2013; Han et al., 2021). During aging in the aorta (Figure 2), NM myosin expression was similar at 6mo (31% ± 2%, n = 6) and 24mo (26% ± 4%, n = 6). However, in the mesenteric artery, the expression of NM myosin significantly decreased with aging. Relative to total myosin expression, NM myosin expression was 22% ± 3% vs. 12% ± 2% (6mo v 24mo, *p* = 0.025).

**FIGURE 3:**
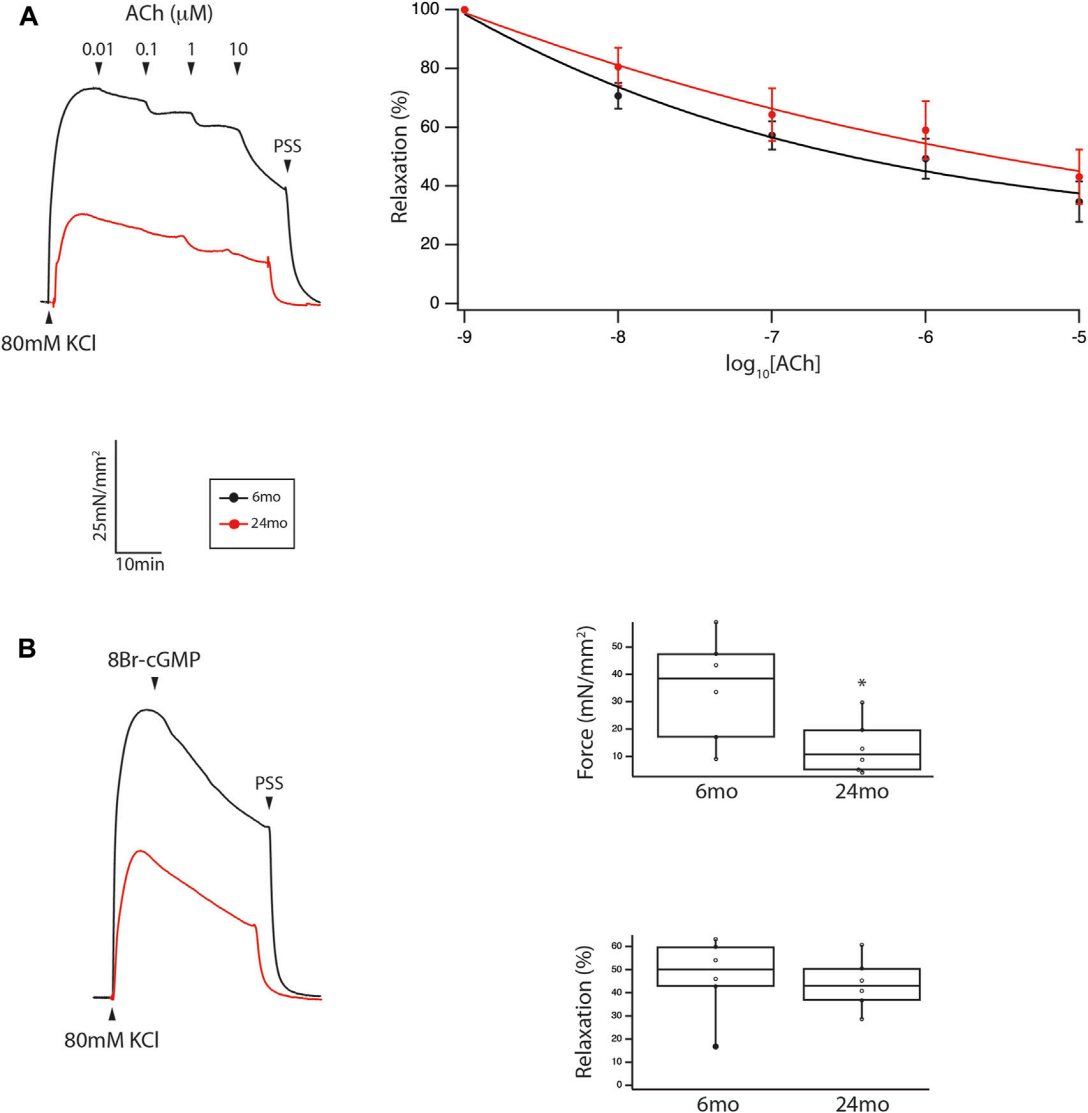
During aging, neither endothelial independent nor endothelial dependent relaxation are altered in a resistance vessel, but force was reduced. **(A)** Force trace demonstrating relaxation to ACh of tertiary mesenteric artery. Sensitivity of ACh mediated relaxation is similar (*p* > 0.05) in mesenteric vessel from young and old animals. **(B)** Relaxation stimulated by 100 μM 8Br-cGMP is no different in tertiary mesenteric arteries from young and old rats (46% ± 7% vs. 43% ± 5%, *p* > 0.05). However, the contraction to 80 mM KCl is significantly depressed in mesenteric vessels from old rats (35 ± 13 mN/mm^2^ vs. 8 ± 4 mN/mm^2^, *p* = 0.016). Box plots summarize the data for both maximal force in response to 80 mM KCl and relaxation to 100 μM 8Br-cGMP (*, *p* < 0.05).

The mechanical properties of tertiary mesenteric arteries were defined by the maximal force in response to 80 mM KCl and the sensitivity of endothelial dependent (ACh) and endothelial independent (8Br-cGMP) relaxation. Force for KCl depolarization was greater at 6mo (n = 6) than 24mo (n = 6); 35 ± 13 mN/mm^2^ vs. 8 ± 4 mN/mm^2^ (Figure 3, *p* = 0.016). Endothelial dependent relaxation was assessed by determining the sensitivity to ACh mediated relaxation; the sensitivity of relaxation to ACh in tertiary mesenteric arteries was similar in vessels isolated from 6mo to 24mo old rats (Figure 3). We also defined endothelial independent relaxation using 8Br-cGMP; 100 μM 8Br-cGMP mediated relaxation was no different in tertiary mesenteric arteries from young and old rats (46% ± 7% vs. 43% ± 5%, *p* > 0.05, Figure 3).

**FIGURE 4:**
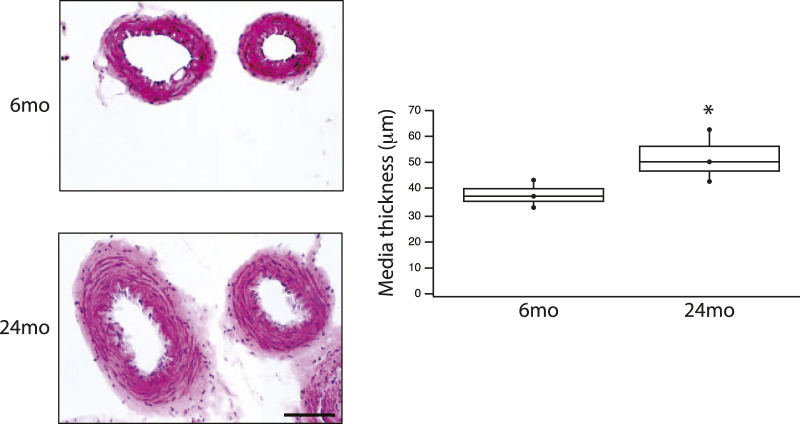
Aging is associated with an increase in media thickness in a resistance vessel. HE stained tertiary mesenteric artery (6mo & 24mo); scale bar, 50 μm (for both images). Box plot summarize the data of the thickness of the media at 6mo (36 ± 3 μm, n = 4) and 24 mo (51 ± 5 μm, n = 4). Aging is associated with a significant (*p* = 0.022) increase in the thickness of the media layer of smooth muscle; *, *p* < 0.05.

**FIGURE 5:**
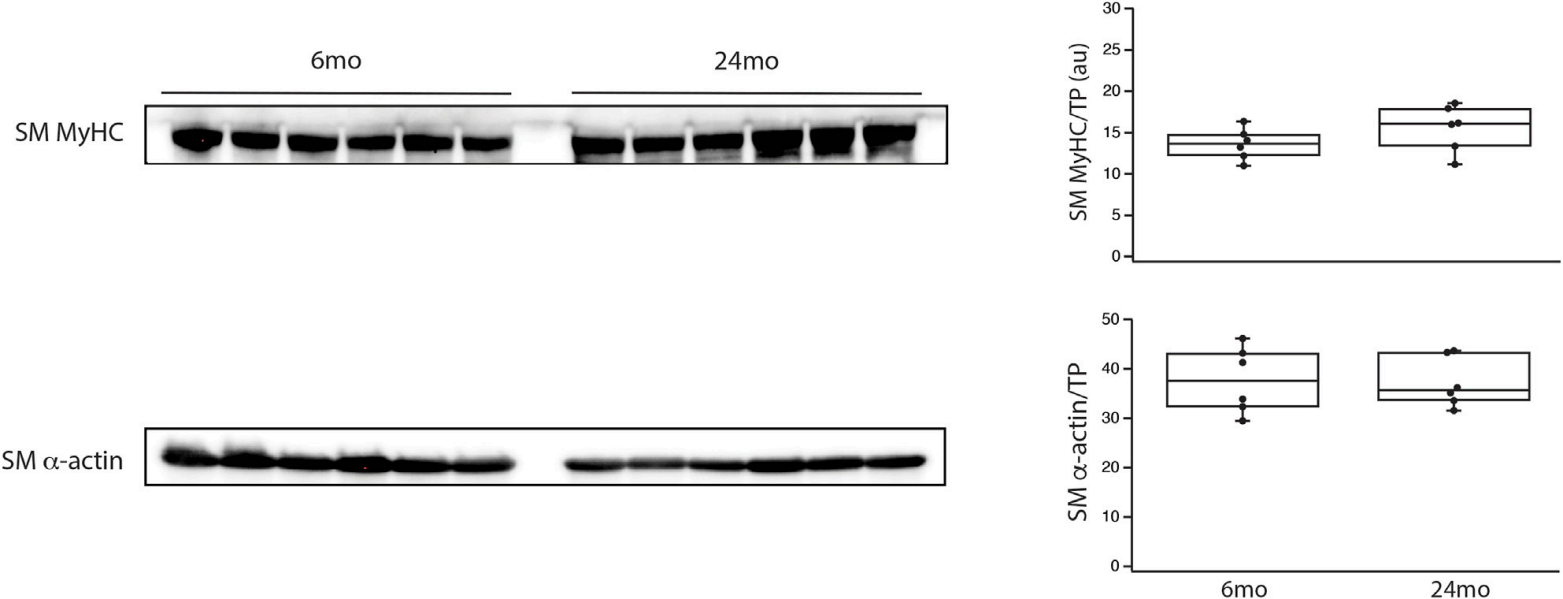
During aging, smooth muscle myosin and α-smooth muscle actin expression did not change. Western blots of smooth muscle myosin heavy chain (SM MyHC) and α-smooth muscle actin (α-SM actin) expression in the aorta and tertiary mesenteric artery. SM MyHC and α-SM actin were normalized to total protein (TP) as previously described (Schaible et al., 2016; Yap et al., 2020; Han et al., 2021). Box blots summarize the data; neither SM MyHC/TP nor α-SM actin/TP expression are altered during aging, *p* > 0.05.

The final series of experiments measured the thickness of the medial smooth muscle layer from stained sections of tertiary mesenteric arteries (Figure 4). Comparing 6mo (n = 4) and 24mo (n = 4), neither lumen diameter (62 ± 8 μm vs. 76 ± 8 μm, *p* > 0.05) nor lumen cross-sectional area (190 ± 20 μm^2^ vs. 240 ± 20 μm^2^, *p* > 0.05) changed. In contrast, the thickness of the medial layer of smooth muscle increased with aging from 36 ± 3 μm at 6mo to 51 ± 5 μm at 24mo (*p* = 0.022, Figure 4). The number of smooth muscle cells in the medial layer of the cross-section was similar (*p* > 0.05); 16 ± 2 (6mo) vs. 17 ± 1 (24mo). Additionally, the expression of both the smooth muscle myosin heavy chain normalized to total protein (SM MyHC/TP) at 6mo (14 ± 1au, n = 6) and 24mo (16 ± 1au, n = 6) and α-smooth muscle actin (α-SM actin/TP) at 6mo (38 ± 2au, n = 6) and 24mo (37 ± 2au, n = 6) was similar (Figure 5, *p* > 0.05).

## Discussion

The results of the present study demonstrate that proteins regulating vascular reactivity are modulated during aging in a resistance vessel (tertiary mesenteric vessel), but not in a conduit vessel (aorta). Vascular tone is regulated by the extent of phosphorylation of MLC_20_ (Barsotti et al., 1987), which is controlled by the activities of MLCK and MLC phosphatase (Hartshorne et al., 1998; Somlyo and Somlyo, 2003; Brozovich et al., 2016). MLCK is regulated by Ca^2+^-calmodulin (Ikebe and Hartshorne, 1985). However, the majority of signaling pathways that regulate vascular tone converge on MLC phosphatase (Hartshorne et al., 1998; Somlyo and Somlyo, 2003; Brozovich et al., 2016). Inhibition of MLC phosphatase (Trinkle-Mulcahy et al., 1995) by Rho kinase (Kitazawa et al., 1991b; 1991a; Swärd et al., 2000; Seko et al., 2003), Zip kinase (MacDonald et al., 2001), ILK (Deng et al., 2001; 2002; Murányi et al., 2002; Wilson et al., 2005), CPI-17 (Eto et al., 1995; 1999; Woodsome et al., 2006), PHI-1 (El-Touhky et al., 2005; El-Toukhy et al., 2006; Eto, 2009) and PKC (Horowitz et al., 1996; Walsh et al., 1996) increases MLC_20_ phosphorylation and vascular tone, while PKG activates MLC phosphatase (Furchgott and Zawadzki, 1980; Lincoln, 1989), which decreases MLC_20_ phosphorylation and vascular tone. Nitric oxide mediated vasodilatation is a fundamental property of the vasculature (Furchgott, 1999), and the sensitivity of NO mediated smooth muscle relaxation has been demonstrated to be dependent on the interaction of PKG with LZ+ MYPT1 (Surks et al., 1999; Given et al., 2007; Sharma et al., 2008). Further, PKG phosphorylates only LZ+ MYPT1 isoforms at S667 (Yuen et al., 2011; 2014), which increases MLC phosphatase activity (Yuen et al., 2014), and consequently, PKG only activates MLC phosphatase expressing a LZ+ MYPT1. Thus, the sensitivity of NO mediated smooth muscle relaxation is dependent on the relative expression of LZ+/LZ- MYPT1 isoforms (Khatri et al., 2001; Huang et al., 2004; Reho et al., 2016). Our data demonstrate that neither MYPT1 nor LZ+ MYPT1 expression changes during aging in either a conduit or a resistance vessel (Figure 1). These results would predict that the sensitivity to both endothelial dependent and endothelial independent vasodilatation would be similar in young and old animals, which is consistent with our results demonstrating that the sensitivity of relaxation to both ACh and 8Br-cGMP is similar in mesenteric vessel of young and old animals (Figure 3). However, a limitation of this study is the use of a single concentration of 8Br-cGMP, which produced similar relaxation of mesenteric vessels from young and old animals (Figure 3), and more concentrations are necessary to completely demonstrate similar sensitivity to 8Br-cGMP. However, our data show no difference in sensitivity to ACh (Figure 3), which would suggest that the sensitivity of 8Br-cGMP should also be similar in tertiary mesenteric arteries of young and old animals.

Endothelial dysfunction is thought to underlie an age-related decrease in NO mediated vasodilatation (Vatner et al., 2021), but there are differences in endothelial function in different vessels as well as location within the same vessel (Vatner et al., 2021). Investigators have demonstrated that the sensitivity of relaxation induced by ACh (endothelial dependent) and nitroprusside (endothelial independent) is impaired in rat abdominal aorta, but not the femoral artery, iliac artery or gastrocnemius muscle artery (Luttrell et al., 2020). However, Barton et al. (Barton et al., 1997) demonstrated that in rats there was an age-related decrease in sensitivity to endothelial dependent, but not endothelial independent relaxation in the aorta, but the response to ACh and nitroprusside was similar in the femoral artery isolated from young and old animals. In mesenteric resistance vessels of elderly mice, compared to young mice, Zhong et al. (Zhong et al., 2021) demonstrated that during aging maximal force and sensitivity to ACh mediated relaxation were reduced, but there was no difference in the sensitivity to nitroprusside. On the other hand, in young mice, Lubomirov et al. (Lubomirov et al., 2018) showed that due to the higher LZ + MYPT1 expression, the basilar artery was more sensitive to both endothelial dependent and endothelial independent relaxation than the femoral artery. Further, the femoral artery isolated from elderly mice, compared to young mice, had a decrease in sensitivity of contraction to thromboxane, and an aging-related decrease in LZ+ MYPT1 expression produced a decrease in sensitivity to ACh (Lubomirov et al., 2021). The relative expression of LZ+/LZ-MYPT1 has been documented to be developmentally regulated (Khatri et al., 2001; Payne et al., 2006), tissue specific (Khatri et al., 2001; Karim et al., 2004; Payne et al., 2004; 2006) and modulated in disease (Karim et al., 2004; Chen et al., 2006; Lu et al., 2008; Ararat and Brozovich, 2009; Han and Brozovich, 2013; Konik et al., 2013; Reho and Fisher, 2015; Lyle et al., 2020; Han et al., 2021). The disparities between our results and those reported by others (Barton et al., 1997; Lubomirov et al., 2016; 2018; 2021; Luttrell et al., 2020; Zhong et al., 2021) could be due to differences in species (rat vs. mouse), strain of animal and/or vessel studied. In addition, MYPT1 has multiple phosphorylation sites which regulate MLC phosphatase activity (Somlyo and Somlyo, 1994; Hartshorne et al., 1998; Brozovich et al., 2016), and in murine basilar arteries, others have demonstrated that MYPT1 phosphorylation at T853 increases during aging (Lubomirov et al., 2023). We did not measure MYPT1 phosphorylation, and changes in MYPT1 phosphorylation could contribute to the disparities among studies. However, our results demonstrate that LZ+ MYPT1 expression does not change with aging (Figure 1). Since the sensitivity to NO mediated vasodilatation is defined by LZ+/LZ- MYPT1 expression (Surks et al., 1999; Huang et al., 2004; Yuen et al., 2011; 2014; Reho et al., 2016), there should be no change in sensitivity to either endothelial dependent or independent smooth muscle relaxation, which agrees with our results (Figure 3). Thus, similar to Lubomirov (Lubomirov et al., 2016; 2018; 2021), our results are consistent with the sensitivity of a vessel to NO being defined by LZ+/LZ- MYPT1 expression; changes in LZ + MYPT1 expression regulate the sensitivity of the vasculature to NO/cGMP/PKG-mediated relaxation (Khatri et al., 2001; Huang et al., 2004; Yuen et al., 2011; 2014; Reho et al., 2016).

In tertiary mesenteric vessels, our data demonstrate that NM myosin expression is significantly lower in elderly compared to young rats (Figure 2). In contrast, in the aorta, NM myosin expression did not change during aging (Figure 2). Similar to SM myosin, NM myosin is regulated by phosphorylation of its light chain (Cremo et al., 2001). Further, NM myosin phosphorylation is regulated during contraction of smooth muscle (Yuen et al., 2009; Zhang and Gunst, 2017) and NM myosin has been demonstrated to participate in force maintenance (Morano et al., 2000; Löfgren et al., 2003; Rhee et al., 2006; Yuen et al., 2009; Zhang and Gunst, 2017; Lubomirov et al., 2023); both a change in NM myosin expression (Morano et al., 2000; Löfgren et al., 2003; Yuen et al., 2009) and inhibition of the NM myosin AMATPase (Rhee and Brozovich, 2003; Lubomirov et al., 2023) have been demonstrated to produce a reduction in force, which demonstrate that changes in NM myosin expression will alter force. In tertiary mesenteric vessels, during aging, our data show NM myosin expression is reduced by ∼10% (Figure 2), while there is no change in NM myosin expression in the aorta, a large conduit vessel. The aging associated decrease in NM myosin expression in tertiary mesenteric vessel would be expected to reduce force (Morano et al., 2000; Löfgren et al., 2003; Rhee et al., 2006; Yuen et al., 2009; Zhang and Gunst, 2017), which is consistent with our results (Figure 3). The mechanism that produces the decrease in NM myosin expression in the tertiary mesenteric vessels with aging is unknown but could be a compensatory mechanism to normalize blood pressure in response to the increase in vascular stiffness documented to occur during aging (Qiu et al., 2010; Vatner et al., 2021).

Aging of the vasculature is associated with an increase in vessel stiffness (Moreau et al., 1998; Barros et al., 2021; Vatner et al., 2021) and histological changes; the ratio of collagen to elastin increases with age (Zhang et al., 2018; Albu et al., 2021; Vatner et al., 2021). Additionally, the ratio of collagen to elastin is higher in the abdominal than the thoracic aorta (Zhang et al., 2018; Vatner et al., 2021), and the increase in collagen/elastin in the vessel contributes to the age-related increase in vessel stiffness (Zhang et al., 2018; Albu et al., 2021; Vatner et al., 2021). Peripheral arteries are less elastic and more muscular, and thus are stiffer than central arteries (Yu and McEniery, 2020), and with aging, the increase in stiffness in peripheral arteries is less pronounced than in the aorta (Yu and McEniery, 2020). In tertiary mesenteric vessels, our data demonstrate neither lumen diameter nor cross-sectional area change during aging. However, the medial layer of smooth muscle of the vessel is significantly thicker in elderly animals (Figure 4), while the cell number is similar (16 ± 2 vs. 17 ± 1, *p* > 0.05) suggesting that smooth muscle cell hypertrophy produces the increase in medial thickness. These data agree with those reported by other investigators that have shown that during aging of rat basilar and mesenteric small arteries, medial thickness increases (Moreau et al., 1998; Loo et al., 2004). However, in mesenteric vessels of mice, others have reported that medial thickness does not change during aging (Zhong et al., 2021). During aging, our data show an increase in the thickness of the media (Figure 4), which would be expected to increase force and vessel stiffness. However, force was lower for KCl stimulated contractions in mesenteric vessels from elderly compared to young rats (Figure 3). The data also demonstrate that SM MyHC and α-SM actin expression does not change with aging (Figure 5), suggesting that the smooth muscle phenotype does not change from contractile to synthetic during aging (Rensen et al., 2007). However, NM myosin has been shown to participate in smooth muscle contraction (Morano et al., 2000; Löfgren et al., 2003; Yuen et al., 2009), and both decreasing NM myosin expression (Yuen et al., 2009) and inhibiting of NM myosin (Rhee et al., 2006; Lubomirov et al., 2023) reduce force. Our data demonstrating a decrease in NM myosin expression in mesenteric arteries (Figure 2) would decrease contractile force, which is consistent with our results (Figure 3).

In summary, our results demonstrate differences in age-related alterations in both the expression of contractile and regulatory proteins in a resistance vs. a conduit vessel, which is consistent with the results of others and demonstrates aging related changes are vessel dependent (Barton et al., 1997; Lubomirov et al., 2018; Luttrell et al., 2020; Vatner et al., 2021). There are no changes in LZ+ MYPT1 or NM myosin in a conduit vessel. In a resistance vessel, our data demonstrate there is no age-related changes in MYPT1 LZ+ expression and consequently, there is no change in the sensitivity to either endothelial dependent (ACh) or endothelial independent (8Br-cGMP) mediated vasodilatation. However, in a resistance vessel, although the thickness of the medial layer of smooth muscle increases with aging, most likely due to hypertrophy of the smooth muscle cells, NM myosin expression is significantly depressed. The decrease in NM myosin expression participates in the mechanism for the aging-related reduction in force in response to KCl depolarization, and the reduction of NM myosin may represent an aging associated compensatory mechanism to mitigate the increase in stiffness of resistance vessels in response to the increase in media thickness and stiffness observed during aging.

## Data Availability

The raw data supporting the conclusion of this article will be made available by the authors, without undue reservation.
